# Suppression of Cholangiocarcinoma Cell Growth and Proliferation by *Atractylodes lancea* (Thunb) DC. through ERK-Signaling Cascade

**DOI:** 10.31557/APJCP.2021.22.11.3633

**Published:** 2021-11

**Authors:** Pongsakorn Martviset, Luxsana Panrit, Pathanin Chantree, Phunuch Muhamad, Kesara Na-Bangchang

**Affiliations:** 1 *Division of Parasitology, Department of Preclinical Science, Faculty of Medicine, Thammasat University, Pathumthani, Thailand. *; 2 *Center of Excellence in Molecular Biology and Pharmacology of Malaria and Cholangiocarcinoma, Thammasat University, Pathumthani, Thailand. *; 3 *Drug Discovery and Development Center, Office of Advanced Science and Technology, Thammasat University, Pathumthani, Thailand. *; 4 *Division of Anatomy, Department of Preclinical Science, Faculty of Medicine, Thammasat University, Pathumthani, Thailand. *; 5 *Research Unit in Nutraceuticals and Food Safety, Faculty of Medicine, Thammasat University, Pathumthani, Thailand. *; 6 *Graduate Program in Bioclinical Sciences, Chulabhorn International College of Medicine, Thammasat University, Pathumthani, 12120, Thailand. *

**Keywords:** Cholangiocarcinoma, Atractylodes lancea, extracellular signal, regulated kinase (ERK), cell proliferation

## Abstract

**Objective:**

The study aimed to investigate the inhibitory effects of AL on the ERK signaling molecules (ERK, p-ERK, cyclin D, and eIF4B) and the growth and proliferation of CCA cells.

**Materials and methods:**

The viability of the three CCA cell lines CL-6, HuCCT1, and HuH28 was determined using MTT assay. The effect of Ras/ERK inhibitors on protein expression in the presence of AL extract was investigated. The protein extracted from each CCA cell following exposure to AL and/or Ras/ERK inhibitors were separated on 12.5% SDS-PAGE. The analysis of mRNA expression following 48 and 72 hours of AL exposure in comparison with 0 hours (non-exposed cells) was performed by using RT-PCR.

**Results:**

The potency of cytotoxic activity of AL (by MTT assay) was about three times higher than the standard drug 5-fluorouracil. The IC_50_ (concentration that inhibits cell growth by 50%) of AL for the CL-6, HuCCT-1 and HuH28 cell lines were 29.77±6.64, 35.45±4.96, and 35.32±6.69 µg/mL (mean+SD), respectively. The cells were exposed to AL extract at the IC_50_ for 0, 12, 24, 48, and 72 hours in the absence and presence of Ras/ERK inhibitors (salirasib and XMD8-92). Protein expression was determined by Western blot analysis. The results suggested the lack of significant inhibitory effect of AL on ERK at 48 and 72 hours of exposure in all CCA cell types. On the other hand, a significant inhibitory effect was observed with p-ERK expression in all CCA cell types. Cyclin D was significantly down-regulated at 72 hours of exposure in all cell types with different potencies. The expression of eIF4B was markedly inhibited in HuCCT-1 but slightly inhibited in CL-6 and HuH28 cells. Real-time PCR analysis revealed significant down-regulation of ERK following 72 hours of AL exposure in the HuCCT1 and HuH28, but not CL-6 cell.

**Conclusion:**

The ERK signaling cascade and downstream molecules are potential targets of action of AL in CCA.

## Introduction

Cholangiocarcinoma (CCA), the biliary ductal cancer, is recognized as a highly progressive cancer that originates from the transformed intra- and extrahepatic cholangiocytes or sometimes metastasis from other adjacent cancers (Alsaleh et al., 2019). High prevalence rates have been reported from several countries, especially in the Mekong subregion, where liver fluke infections (*Opisthorchis viverrini, Opisthorchis felineus, and Clonorchis sinensis*) are endemic (Kirstein and Vogel, 2016). Long-term parasitic infection, coupled with the consumption of the precarcinogen dimethyl-nitrosamine (DMN) in the fermented fish and meats, is the major risk factor of CCA in this area (Sripa et al., 2007). Other risk factors include primary sclerosing cholangitis (PSC), hepatolithiasis, drug usage, and genetic factor (Khan et al., 2005; Lazaridis and Gores, 2005). The prognosis of CCA patients is generally poor due to the rapid and aggressive cell proliferation. The most effective treatment is surgery, but the prognosis is markedly variable (Charonpongsuntorn et al., 2019). The average survival time ranges from 6 to 12 months (Ustundag and Bayraktar, 2008). Effective control of CCA has been limited by the lack of specific and sensitive diagnostic tools for early detection of CCA as well as effective chemotherapeutic drugs (Ustundag and Baraktar, 2008). 

*Atractyloides lancea* (Thunb.) DC. (AL), a tuber plant belonging to the family Asteraceae (Compositae), distributes in several Asian countries, such as China, Japan, and Korea. The dried rhizome has long been used for the treatment of several illnesses and health conditions such as rheumatic fever, digestive diseases, night-blindness syndrome, fever, and cold (Na-Bangchang and Karbwang, 2014; Jun et al., 2018). The plant rhizome extract has been shown in the* in vitro* and *in vivo* models to exert anticancer, anti-inflammatory, antibacterial, and antifungal activities. Besides, it also possesses antipyretic, analgesic, and immunomodulatory activities (Koonrungsesomboon et al., 2014). The anti-CCA potential of AL and its major constituents (e.g. β-eudesmol and atractylodin) have recently been reported in a series of non-clinical studies (Na-Bangchang et al., 2017). The antiproliferative activity, inhibitory activities on cell migration and cell invasion, inducing effect on cell cycle arrest and apoptosis are well demonstrated (Kotawong et al., 2018; Methema et al., 2015). Nevertheless, the intracellular signaling cascades of AL action remain to be elucidated. 

Excessive cell proliferation is one of the most critical properties of cancerous cells. It is triggered by several stimuli, leading to the alteration of several downstream signaling molecules to promote cell proliferation. The most investigated cell proliferation cascade is Ras/Raf/ERK (extracellular signal-regulated kinase) signaling pathway (Sever and Brugge, 2015). This cascade is stimulated by extracellular ligands such as mitogens, serum, growth factors, cytokines, stress, GPCRs (G-protein coupled receptors) ligands, and calcium ions (Lavoie et al., 2020). Mutations of the proto-oncogenes and tumor suppressor genes or inappropriate synthesis of ligands and receptors can hyperactivate many signaling pathways, leading to activation of the cell cycle machinery. Activated-ERK phosphorylates numerous downstream molecules, either enhancing cell proliferation, e.g., Myc, RSK (90 kDa ribosomal S6 kinase), MNK, and Fos-Jun), or inhibiting tumor-suppressive molecules such as CKIs (cyclin-dependent kinase inhibitors). The phosphorylated Myc stimulates cell proliferation by activating numerous genes, including those encoding CyclinD/E, CDK4/6 (cyclin-dependent kinase4/6), and E2F-family transcription factors (Sever and Brugge, 2015). Multiple kinases in the mitogen-, stress-activated kinase (MSK), and ribosomal S6 kinase (RSK) are also phosphorylated by ERK, and these kinases, in turn, phosphorylate the transcription factors that regulate cell cycle progression (Roux and Blenis, 2004). MSKs play a crucial role in regulating translation following mitogenic stimulation by phosphorylating the translation initiation factor eIF4E (Soloaga et al., 2003). RSK regulates gene translation by phosphorylating eIF4B, which increases its interaction with the translation initiation factor eIF3. Moreover, eIF4B is also activated directly by the phosphorylated ERK. Activation of the translation process by these mechanisms is essential for cell growth and proliferation (Sever and Brugge, 2015). In CCA, ERK signaling cascade has been documented as important cancer surviving pathway (Schmitz et al., 2007). In this study, the effects of AL rhizome extract on cell growth and proliferation associated with ERK signaling pathway were investigated in the three CCA cell lines, i.e., CL-6, HuCCT1, and HuHT28 cells. The effects of ERK/Ras inhibitors on modulating AL action was also examined.

## Materials and Methods

*Preparation of ethanolic AL rhizome crude extract*

The crude ethanolic extract of AL was prepared as previously described (Plengsuriyakarn et al., 2015). Briefly, the rhizomes were washed with tap water, cut into small pieces, and dried in the hot air oven (50 ºC) for 2-3 days. The dried AL rhizomes were powdered using an electric grinder, and 100 g of the powder was transferred to a stoppered flask containing 95% ethanol and incubated at room temperature (25-30 ºC) for seven days. The extract was filtered through Whatman^®^ No. 1 filter paper (Sigma-Aldrich, Darmstadt, Germany), evaporated in the rotary evaporator (IKA Rotary evaporator RV 3 V-C, Staufen, Germany), and stored at -20ºC until use.

*Cell culture*

The CCA cell lines used in the study were CL-6, HuCCT1, and HuH28. CL-6 cell was obtained directly from Thai patients and was established and kindly provided by Associate Professor Adisak Wongkajornsilp, Department of Pharmacology, Faculty of Medicine Sirisaj Hospital, Mahidol University, Thailand. HuCCT1 cell was obtained from the Japanese Collection of Research Bioresources Cell Bank (JCRB cell bank, Osaka, Japan). HuH28 cell was obtained from the JCRB cell bank. All were cultured in RPMI-1640 medium supplemented with 10% fetal bovine serum and 1% Antibiotic-Antimycotic (Gibco™, ThermoFisher Scientific, Rockford, USA) at 37ºC under 5% CO_2_. The cells were cultured until reaching approximately 80% confluence and detached from primary culture flasks by treating with 0.25% trypsin-EDTA (Gibco™) for 1-2 minutes. The cells were stained with 0.4% trypan blue (Sigma-Aldrich, Darmstadt, Germany), and cell number was counted using a hemocytometer.

*Concentration-response analysis*

The viability of the three CCA cell lines following exposure to various concentrations of AL extract (200, 100, 50, 25, 12.5, 6.25, 3.13, and 1.56 µg/mL) was determined using MTT assay (Sigma-Aldrich, Mannheim, Germany). In brief, the cells were seeded onto a 96-well microtiter plate (1×10^4^ cells/well) and incubated at 37ºC under 5% CO_2 _for 24 hours. Cell quality was examined under an inverted light microscope. 5-Fluorouracil (5-FU) was used as a reference compound (two-fold serial dilution started from 1,000 µg/mL). The extract and 5-FU were added to each well, and the plate was further incubated for 48 hours. Cell viability was determined by adding 10 µL of MTT substrate and incubated at 37ºC under 5% CO_2_ for 4 hours. The cells were dissolved in DMSO, and the absorbance was measured at the wavelength of 570 nm. The experiment was performed in three independent assays, triplicate each. The IC_50_ (concentration that inhibits cell growth by 50%) was calculated using the concentration-response analysis software CalcuSynTM (Biosoft, Cambridge, UK). 

*Effect of incubation time on CCA cell growth in the presence of AL exposure*

CL-6, HuCCT1, and HuH28 were plated onto 96-well microtiter plates (1×10^4^ cells/well) and incubated at 37ºC under 5% CO_2_ for 24 hours. AL extract at the IC_50_ concentration was added to each well, and cell viability at 0, 12, 24, 48, and 72 hours was measured by MTT assay as described above. Non-exposed cells served as control of normal cell growth. The experiment was performed in three independent assays, triplicate each. 

*Exposure of CCA cells to AL and Ras/ERK inhibitors*

The AL-exposed and non-exposed cells (CL-6, HuCCT1, and HuH28) were cultured in 25 cm^2^ culture flasks at the density of 1×10^6^ cells at 37 ºC under 5% CO_2_ atmosphere for 24 hours. The cells were exposed to AL extract in 50% ethanol at the IC_50_ concentration for 12, 24, 48, and 72 hours. Culture medium was removed, and the cells were washed three times with cold PBS (pH 7.4). Total proteins were extracted using RIPA buffer (Sigma-Aldrich, Mannheim, Germany) containing protease inhibitors (Merck Millipore Calbiochem™ Protease Inhibitor Cocktail Set III, EDTA-Free, Darmstadt, Germany), and the concentrations were measured by BCA assay (Pierce^TM^ BCA protein assay kit, Thermo Scientific, Rockford, USA). 

The effect of Ras/ERK inhibitors on protein expression in the presence of AL extract was further investigated in the three CCA cell lines. Each cell line (1×10^6^ cells) was seeded onto a 25 cm2 culture flask and incubated at 37 ºC under 5% CO_2_ for 24 hours. The cells were exposed to AL in the presence and absence of the ERK inhibitor (XMD8-92), Ras inhibitor (salirasib) as follows: control cells (without AL, XMD 8-92, or salirasib); AL (2 x IC50) alone; XMD 8-92 (0.5 µM) alone; salirasib (0.5 µM) alone; AL (2 x IC50) + XMD 8-92 (0.5 µM); and AL (2 x IC_50_) + salirasib (0.5 µM). At the end of 72 hours of exposure (37 ºC, 5% CO_2_), proteins were extracted using RIPA buffer and concentrations measured by BCA assay. The experiments were performed in three independent assays, triplicate each.

*Western blot analysis*

The protein (10 µg) extracted from each CCA cell following exposure to AL and/or Ras/ERK inhibitors were separated on 12.5% SDS-PAGE (Bio-rad Laboratories Inc., Hercules, USA). Separated protein bands were transferred to the nitrocellulose membrane (Hybond ECL membrane, GE AmershamTM, Buckinghamshire, UK) using a semi-dry transfer (Trans-Blot^®^ TurboTM transfer system, Bio-rad Laboratories Inc., Hercules, USA). The membranes were stained with 0.1% Ponceau S dye and washed three times with TBS (pH 7.5) to remove the remaining dye. Non-specific binding was blocked using 4% BSA (Sigma-Aldrich, St. Louis, USA) in TBS (pH 7.5) for one hour at room temperature with agitation. Primary antibodies used in the experiments were 1:1,000 anti-ERK1/2 (rabbit p44/42 [ERK1/2], Cell Signaling Technologies Inc., Beverly, USA), 1:1,000 anti-phospho-ERK1/2 (rabbit phospho-p44/42 [ERK1/2] [Thr202/Tyr204], Cell Signaling Technologies Inc.), 1:2,000 anti-cyclin D1 [EPR2241] - C-terminal (Abcam, Cambridge, USA), 1:2,000 anti-eIF4B (Abcam), and 1:1,000 anti-β-actin (Enzo Life Sciences Inc., Farmingdale, USA). Each antibody was diluted in TBS (pH 7.5) containing 1% BSA and added to the membranes and incubated overnight at 4 ºC with gentle shaking. The membranes were washed with TBS (pH 7.5) containing 0.05% Tween™-20 three times. Secondary antibodies, i.e., 1:30,000 goat anti-rabbit IgG H&L AP-conjugated (Abcam) and 1: 30,000 goat anti-mouse IgG H&L AP-conjugated (Abcam) were added onto the membranes and incubated at room temperature for one hour with gentle shaking. The membranes were washed and immersed in detection buffer (pH 9.5) for 5 min. BCIP/NBT substrate (VWR life Sciences, Radnor, USA) was added, and the membranes were incubated at room temperature in the dark without shaking until the positive signal was observed. Densitometric analysis of the positive bands was performed using ImageJ software (imagej.nih.gov).

*Semi-quantitative real-time polymerase chain reaction*

The analysis of mRNA expression following 48 and 72 hours of AL exposure in comparison with 0 hours (non-exposed cells) was performed using RT-PCR. Each CCA cell (5×10^5^ cells) were seeded onto a 6-well culture plate and incubated at 37 ºC under 5% CO_2_ for 24 hours. The cells were exposed to AL extract in 50% ethanol at the IC_50_ concentration for 48 and 72 hours. At the end of each incubation time, culture medium was removed, and the cells were washed three times with cold PBS (pH 7.4). TRIzol^TM^ reagent (Invitrogen, Thermo Scientific Inc., Carlsbad, CA, USA) was added, and total RNA content was isolated according to the manufacturer’s protocol. The RNA was converted to complementary DNA (cDNA) using a cDNA synthesis kit (RevertAid First Strand cDNA Synthesis kit, Thermo Fisher Scientific, St. Louis, USA) with Oligo (dT) 18 primer. The cDNA concentration was quantified using NanoDrop^TM^ 2000 spectrophotometers (Thermo Fisher Scientific, Wilmington, DE, USA). An aliquot of 100 ng cDNA was used as a template for RT-PCR analysis PCR using iTaq™ Universal SYBR^®^ Green Supermix (Bio-rad Laboratories Inc.) in a CFX96™ Real-Time PCR machine (Bio-rad Laboratories Inc., Hercules, CA, USA). The mRNA expression of ERK1/2 was determined and normalized with GAPDH (an internal control) for semi-quantification of the PCR products. The primers used were ERK1/2 (F) 5´-TCAAGCCTTCCAACCTC-3´, ERK1/2 (R) 5´- GCAGCCCACAGACCAAA-3´, GAPDH (F) 5´-AGCCACATCGCTCAGACA-3´, and GAPDH (R) 5´- TGGACTCCACGACGTACT-3´. The PCR condition consisted of denaturation at 95°C for 1 min and amplification for 40 cycles at the following conditions: 95°C for 30 sec, 50°C for 30 sec, and 72°C for 2 min, and followed by final heating at 72°C for 3 min. The fold change of the gene expression level was determined using the 2^∆∆CT^ method (Schmittgen and Livak, 2008). The experiments were performed in three independent assays, triplicate each. 

*Statistical analysis*

The statistical analysis was performed using SPSS version 17 (IBM, Armonk, USA). Data are expressed as mean±SD values. Comparison of more than two quantitative data sets was performed using ANOVA. Comparison of the two quantitative data sets was performed using an independent t-test or paired t-test where appropriate. The statistical significance was set at α = 0.05. 

## Results

*Antiproliferative activity of AL extract against CCA cell lines*

The IC_50_ (concentration that inhibits cell growth by 50%) values (mean ± SD) of AL extract for the CL-6, HuCCT1, and HuH28 were 29.77± 6.64, 35.45±4.96, and 35.32±6.69 µg/mL, respectively. The corresponding IC_50_ values of the reference drug (5-FU) were 94.05±10.74, 93.26±6.35, and 104.01±11.42 µg/mL, respectively. 

*Inhibitory effect of AL extract on CCA cells growth*

**Figure 1:**
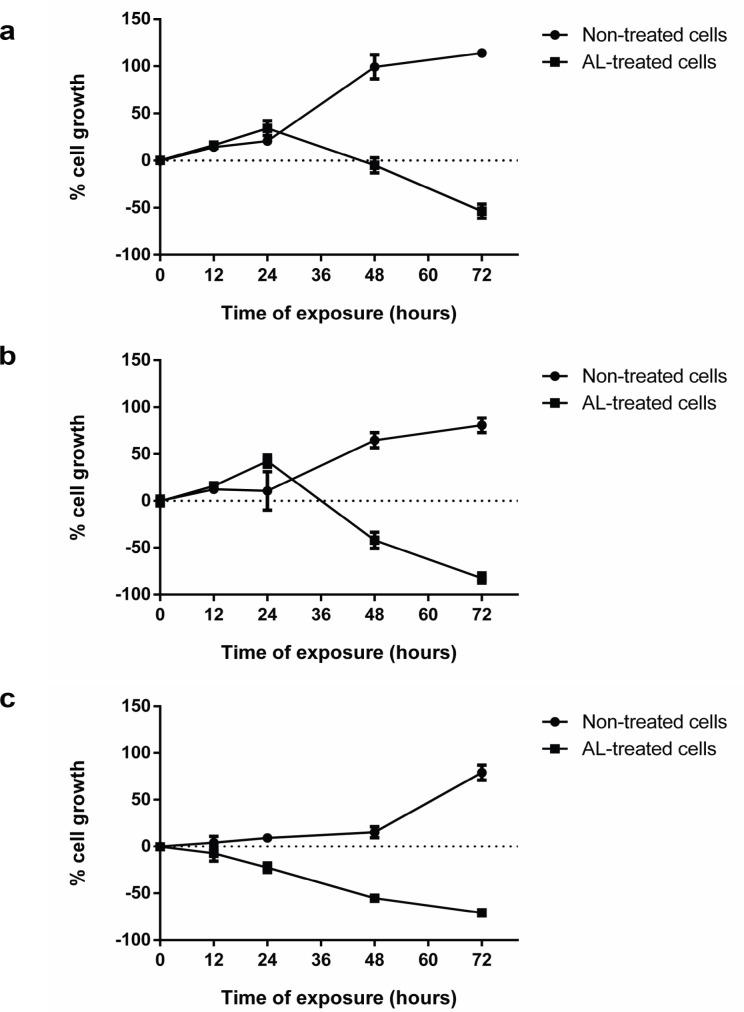
The Growth of CCA Cells (% of Baseline) Following Exposure to AL Extract and Non-Exposed Control Evaluated by MTT Assay: (a) CL-6, (b) HuCCT1, and (c) HuH28. Data are presented as mean ± SD values

The growth of CL-6 and HuCCT1 cells showed similar patterns during the 72 hours, characterized by the rapid growth phase during the first 48 hours, followed thereafter, by the relatively slow growth phase. In the presence of AL extract, the slow growth phase was observed during the first 24 hours but significantly inhibited after 24 hours. The growth of both CCA cells was markedly inhibited (60-90%) at 72 hours of exposure (Figure 1a, b). The HuH28 cell, on the other hand, showed a relatively slow growth phase during the first 48 hours, followed by a sharp growth phase. In the presence of AL extract, cell growth was gradually inhibited until 72 hours (Figure 1c). 

*Inhibitory effect of AL extract on the expression of downstream ERK-stimulated proteins*

**Figure 2:**
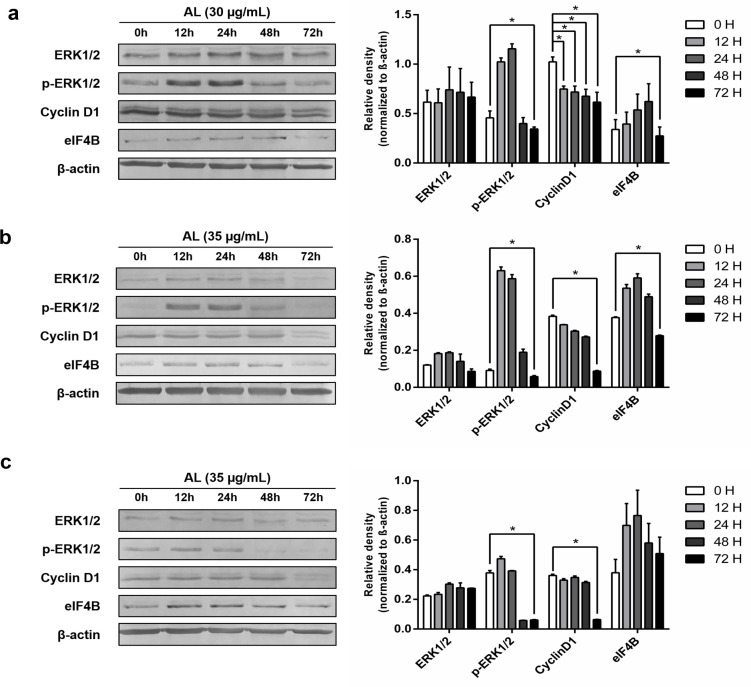
Western Analysis of ERK1/2 and Downstream Proteins, p-ERK1/2, cyclin D1, and eIF4B, Following Exposure to AL Extract: (a) CL-6, (b) HuCCT1, and (c) HuH28. * (p < 0.01) represents statisticaly significant difference (independent t-test or paired t-test).

The inhibitory effects of AL on the level of p-ERK1/2, cyclin D1, and eIF4B proteins are shown in Figure 2 (a, b, c). Results of the western analysis revealed significant down-regulation of p-ERK1/2 level in all of the three CCA cell lines at almost all AL exposure periods compared with baseline (0 hour). In addition, significant down-regulation was also observed at 48 and 72 hours compared with 12 and 24 hours of exposure. The expression of cyclin D1 in all cell lines was, however, significantly down-regulated only at 72 hours compared with baseline. For eIF4B, the expression in HuCCT1 was significantly down-regulated up to 72 hours of exposure. Significant down-regulation in the expression of the HuH28 cells was observed as early as 12-24 hours, while that of the CL-6 cell was delayed until 48 and 72 hours of exposure. 

*Inhibitory effect of AL extract on the expression of downstream ERK-stimulated proteins in the presence of Ras/ERK inhibitors*

**Figure 3:**
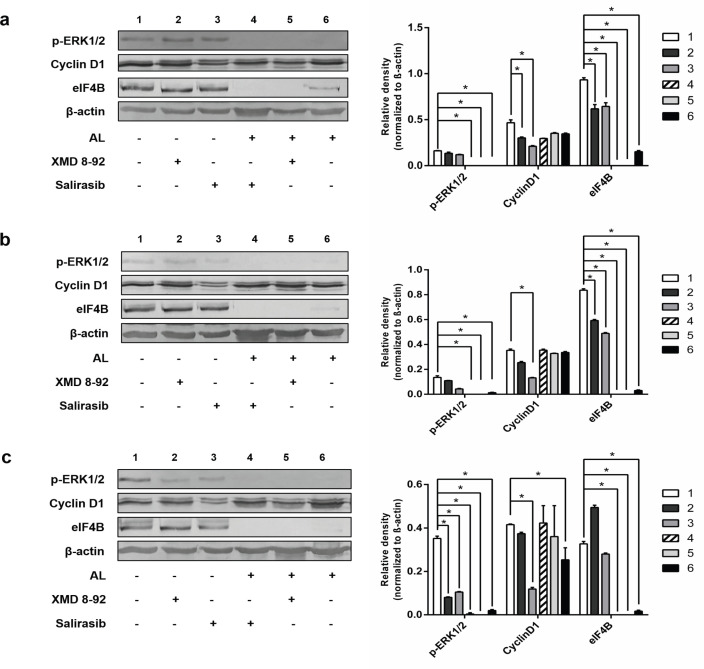
Western Analyses of the Three CCA Cell Lines Cells Following Exposure to AL Alone, XMD8-92 Alone, Salirasib Alone, AL in Combination with XMD8-92, and AL in Combination with Salirasib: (a) CL-6, (b) HuCCT1, and (c) HuH28. * (p < 0.01) represents statistically significant difference (independent t-test or paired t-test)

The inhibitory effects of AL extract, XMD8-92 (ERK inhibitor), and salirasib (Ras inhibitor) on p-ERK1/2 level in CL-6, HuCCT1, and HuH28 cells are shown in Figure 3 (a, b, c). The level of p-ERK1/2 in HuH28 cells was significantly down-regulated when exposed to AL alone, XMD8-92 alone, salirasib alone, and in particular, AL in combination with XMD8-92 or salirasib. For CL-6 and HuCCT1 cells, significant down-regulation was observed only with AL alone, and AL in combination with XMD8-92 or salirasib.

The expression of cyclin D1 was significantly down-regulated after exposing all CCA cell lines to salirasib. On the other hand, its expression was down-regulated after exposing HuH28 and CL-6 cell lines to AL or XMD8-92. For eIF4B, the expression was down-regulated in CL-6, and HuCCT1 cells following exposure to AL alone, XMD8-92 alone, salirasib alone, and in particular AL in combination with XMD8-92 or salirasib. In HuH28 cells, the expression was significantly down-regulated only when exposing to AL extract alone, and AL extract in combination with XMD8-92 or salirasib.

*Effect of AL extract on EKR mRNA expression*

**Figure 4:**
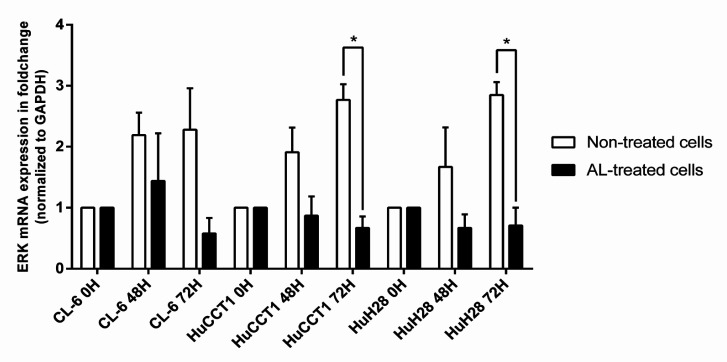
Semi-Quantitative Real-Time PCR of ERK1/2 Expression in CL-6, HuCCT1, and HuH28 Significant Difference (Independent t-test or Paired t-test)

Results of the semi-quantitative RT-PCR analysis showed that the mRNA expression of ERK normalized with GAPDH in the AL-exposed and non-exposed CL-6 cells were comparable at 48- and 72-hours exposing periods. The expression in HuCCT1 and HuH28 cells were significantly down-regulated after 72 hours of exposure (Figure 4).

## Discussion

The results of the present study confirmed the potent inhibitory activity of AL extract (about 3-fold of 5-FU) on the proliferation of different types of CCA cell lines of the intrahepatic and extrahepatic origins. The observed IC_50_ values for the CL-6, HuCCT1, and HuH28 were in agreement with that previously reported in CCA from both ethanolic crude extract and bioactive constituents (Plengsuriyakarn et al., 2012; Mahavorasirikul et al., 2010; Martviset et al., 2018; Kotawong et al., 2018; Mathema et al., 2017). In all CCA cell lines, significant cell growth inhibition was observed at 48 and 72 hours of exposure. The time-dependent activity of AL extract suggested its action on cell growth early before (0-24 hours) as well as after (24-72 hours) cell division. This may suggest that the dose regimens of the AL extract should be adequately prolonged to eliminate all CCA cells. The previous study in animal models demonstrated a significant reduction of CCA tumor mass following chronic dosing (Plengsuriyakarn et al., 2015). 

Ras/Raf/ERK pathway is the primary pathway involved in cell proliferation control. ERK is one of the most critical molecules responsible for several cell behaviors, especially cell growth and cell proliferation (Sever and Brugge, 2015). In cancer cells, excessive ERK activation leads to uncontrolled cell division and abnormal cell proliferation (Lavoie et al., 2020). Our results suggested that the antiproliferative activity of the AL extract was through ERK cascade. The levels of p-ERK in all the three CCA cell lines were significantly reduced after 24-48 hours of exposure to AL. This is the period of highest cell proliferation (Lavoie et al., 2020). p-ERK normally activates several downstream molecules involved in cell growth, particularly transcription and initiation factors including cyclin D1 and eIF4B. Cyclin D1 regulates cell division in the G1/S phase transition, which can be stimulated by several molecules, including Myc that is upwardly activated by p-ERK (Sears et al., 2000). Low level of p-ERK results in the reduction of Myc and finally, the cyclin D1 level. AL was shown to reduce p-ERK and cyclin D1 production in all of the three CCA cell lines. This resulted in the termination of cell division which coincided with its inhibitory effect on CCA cell proliferation. eIF4B is another initiation factor that can be directly stimulated by ERK or indirectly activated through the ERK-RSK pathway. It is incorporated with eIF4A and binds to the eIF3 complex, leading to the recruitment of the small ribosomal subunit (40s) to the mRNA, which in turn, results in protein synthesis necessary for cell growth (Soloaga et al., 2003). AL significantly reduced the level of eIF4B in HuCCT1, but not in other cell lines where a trend of decreasing expression was observed during the 12-72 hours of exposure. Altogether, the findings suggested that the action of AL on the signaling molecules in the ERK cascade may be different in various cell types depending on CCA cell origins. The three CCA cell lines used in the experiments exhibit different pathological, biochemical, and genetic characteristics. The CL-6 cell line is a highly proliferative intrahepatic CCA cell line, whereas the HuCCT1 cell line is a moderately differentiated intrahepatic CCA cell. The HuH28 is a slow growth CCA cell that is originated from the gallbladder. 

The present study is the first study that investigated the modulatory effects of ERK/Ras inhibitors, XMD8-92, and salirasib, on protein and mRNA expression of p-ERK, cyclin D1, and eIF4B in CCA cell lines. Salirasib, the Ras inhibitor, has been used to investigate the potential molecular targets of various compounds in cancer, especially those with KRAS mutation (Furuse et al., 2018). XMD8-92 effectively inhibits ERK undefinably pathway (Chin et al., 2019; Sureban et al., 2014). In this study, salirasib or XMD8-92 alone was shown to inhibit p-ERK and cyclin D1, but not eIF4B. The combination of either inhibitor with AL, however, produced complete suppression of the expression of p-ERK and eIF4B proteins. The significant down-regulation of cyclin D1 level in all three CCA cell lines exposing to salirasib alone suggested that salirasib could affect cyclin D1 expression in CCA cells as reported in other cancer cells (Charette et al., 2010). In contrast, the cyclin D1 levels in the cells exposed to AL in combination with salirasib were comparable with that of the untreated cells. This result suggested the antagonistic effect between AL and salirasib. The underlying mechanism needs further investigation. The inhibitory effect of AL extract on the expression of ERK was confirmed by the results of mRNA expression using semi-quantitative RT-PCR. The effect was prominent at 72 h of exposure in all cell lines, but statistical significance was observed only in HuCCT1 and HuH28 cell lines. It was noted however that ERK mRNA expression in CL-6 cell was decreased at 72 h when compared with non-treated control cells. 

In conclusion, the results of the study confirm that AL exerts significant inhibition of the proliferation of all CCA cell types through intracellular ERK cascade, which is potentiated by ERR/Ras inhibitors. A thorough understanding of the molecular mechanism of AL, especially cell growth and proliferation, is essential for the further development of this plant for CCA control.

## Author Contribution Statement

PM and KN were involved in the design of the experimental study. PM, LP, PC and PM performed the experiments and data analysis. PM drafted the manuscript. KN revised the manuscript. All authors reviewed and approved the final manuscript for submission. All meet the ICMJE criteria for authorship.

## Data availability statement

All data used to support the findings of this study are available from the corresponding author upon request.

## Conflict of interest

The authors declare that there is no conflict of interest regarding the publication of this paper.

## References

[B1] Alsaleh M, Leftley Z, Barbera TA (2019). Cholangiocarcinoma: a guide for the nonspecialist. Int J Gen Med.

[B2] Chanchai C, Piyasatit P, Muntham D, Chommaitree P, Muangnoi P (2019). Clinical prognostic factors and treatment Outcomes for the survival of patients with cholangiocarcinoma in the Eastern Region of Thailand. Asian Pac J Cancer Cares.

[B3] Charette N, De Saeger C, Lannoy V (2010). Salirasib inhibits the growth of hepatocarcinoma cell lines in vitro and tumor growth in vivo through ras and mTOR inhibition. Mol Cancer.

[B4] Chin HM, Lai1 DK, Falchook GS (2019). Extracellular signal-regulated kinase (ERK) inhibitors in oncology clinical trials. J Immunother Prec Oncol.

[B5] Furuse J, Kurata T, Okano N (2018). An early clinical trial of salirasib, an oral RAS inhibitor, in Japanese patients with relapsed/refractory solid tumors. Cancer Chemother Pharmacol.

[B6] Jun X, Fu P, Lei Y, Cheng P (2018). Pharmacological effects of medicinal components of Atractylodes lancea (Thunb) DC. Chin Med.

[B7] Khan SA, Thomas HC, Davidson BR, Taylor-Robinson SD (2005). Cholangiocarcinoma. Lancet.

[B8] Kirstein MM, Vogel A (2016). Epidemiology and risk factors of cholangiocarcinoma. Vis Med.

[B9] Koonrungsesomboon N, Na-Bangchang K, Karbwang J (2014). Therapeutic potential and pharmacological activities of Atractylodes lancea (Thunb) DC. Asian Pac J Trop Med.

[B10] Kotawong K, Chaijaroenkul W, Muhamad P, Na-Bangchang K (2018). Cytotoxic activities and effects of atractylodin and β-eudesmol on the cell cycle arrest and apoptosis on cholangiocarcinoma cell line. J Pharmacol Sci.

[B11] Lavoie H, Gagnon J, Therrien M (2020). ERK signaling: a master regulator of cell behaviour, life and fate. Nature Rev Mol Cell Biol.

[B12] Lazaridis KN, Gores GJ (2005). Cholangiocarcinoma. Gastroenterol.

[B13] Mahavorasirikul W, Viyanant V, Chaijaroenkul W, Itharat A, Na-Bangchang K (2010). Cytotoxic activity of Thai medicinal plants against human cholangiocarcinoma, laryngeal and hepatocarcinoma cells in vitro. BMC Complement Altern Med.

[B14] Martviset P, Chaijaroenkul W, Muhamad P, Na-Bangchang K (2018). Bioactive constituents isolated from Atractylodes lancea (Thunb ) DC rhizome exhibit synergistic effect against cholangiocarcinoma cell. J Exp Pharmacol.

[B15] Mathema VB, Chaijaroenkul W, Karbwang J, Na-Bangchang K (2017). Growth inhibitory effect of β-eudesmol on cholangiocarcinoma cells and its potential suppressive effect on heme oxygenase-1 production, STAT1/3 activation, and NF-κB downregulation. Clin Exp Pharmacol Physiol.

[B16] Mathema VB, Chaijaroenkul W, Na-Bangchang K (2019). Cytotoxic activity and molecular targets of atractylodin in cholangiocarcinoma cells. J Pharm Pharmacol.

[B17] Na-Bangchang K, Karbwang J (2014). Traditional herbal medicine for the control of tropical diseases. Trop Med Hlth.

[B18] Na-Bangchang K, Plengsuriyakarn T, Karbwang J (2017). Research and development of Atractylodes lancea (Thunb) DC as a promising candidate for cholangiocarcinoma chemotherapeutics. Evid-Based Compl Alt Med.

[B19] Plengsuriyakarn T, Matsuda N, Karbwang J (2015). Anticancer activity of Atractylodes lancea (Thunb) DC in a hamster model and application of PET-CT for early detection and monitoring progression of cholangiocarcinoma. Asian Pac J Cancer Prev.

[B20] Plengsuriyakarn T, Viyanant V, Eursitthichai V, Itharat A, Na-Bangchang K (2012). In vitro investigations on the potential roles of Thai medicinal plants in treatment of cholangiocarcinoma. Int Res J Pharm Pharmacol.

[B21] Roux PP, Blenis J (2004). ERK and p38 MAPK-activated protein kinases: A family of protein kinases with diverse biological functions. Microb Mol Biol Rev.

[B22] Schmittgen TD, Livak KJ (2008). Analyzing real-time PCR data by the comparative CT method. Nat Proto.

[B23] Schmitz KJ, Lang H, Wohlsschlaeger J (2007). AKT and ERK1/2 signaling in intrahepatic cholangiocarcinoma. World J Gastroenterol.

[B24] Sears R, Nuckolls F, Haura E (2000). Multiple Ras-dependent phosphorylation pathways regulate Myc protein stability. Genes Develop.

[B25] Sever R, Brugge JS (2015). Signal transduction in cancer. Cold Spr Harb Pers Med.

[B26] Soloaga A, Thomson S, Wiggin GR (2003). MSK2 and MSK1 mediate the mitogen- and stress-induced phosphorylation of histone H3 and HMG-14. Eur Mol Biol Organ J.

[B27] Sripa B, Kaewkes S, Sithithaworn P (2007). Liver fluke induces cholangiocarcinoma. PLos Med.

[B28] Sureban SM, May R, Weygant N (2014). XMD8-92 inhibits pancreatic tumor xenograft growth via a DCLK1-dependent mechanism. Cancer Lett.

[B29] Ustundag Y, Bayraktar Y (2008). Cholangiocarcinoma: a compact review of the literature. World J Gastroenterol.

